# The Conserved Arginine Cluster in the Insert of the Third Cytoplasmic Loop of the Long Form of the D_2_ Dopamine Receptor (D_2L_-R) Acts as an Intracellular Retention Signal

**DOI:** 10.3390/ijms17071152

**Published:** 2016-07-19

**Authors:** Valentina Kubale, Kaja Blagotinšek, Jane Nøhr, Karin A. Eidne, Milka Vrecl

**Affiliations:** 1 Institute of Anatomy, Histology & Embryology, Veterinary Faculty, University of Ljubljana, Gerbičeva 60, SI-1000 Ljubljana, Slovenia; valentina.kubale@vf.uni-lj.si (V.K.); kaja1.blagotinsek@gmail.com (K.B.); 2 Department of Incretin & Islet Biology, Novo Nordisk A/S, DK-2760 Måløv, Denmark; jnql@novonordisk.com; 3 Laboratory for Molecular Endocrinology-G Protein-Coupled Receptors, Western Australian Institute for Medical Research (WAIMR) and Centre for Medical Research, The University of Western Australia, WA 6009 Perth, Australia; keidne@gmail.com

**Keywords:** D_2_ dopamine receptors, endoplasmic reticulum (ER) retention motif, confocal microscopy, surface expression, bioluminescence resonance energy transfer (BRET^2^), cAMP signaling

## Abstract

This study examined whether the conserved arginine cluster present within the 29-amino acid insert of the long form of the D_2_ dopamine receptor (D_2L_-R) confers its predominant intracellular localization. We hypothesized that the conserved arginine cluster (RRR) located within the insert could act as an RXR-type endoplasmic reticulum (ER) retention signal. Arginine residues (R) within the cluster at positions 267, 268, and 269 were charge-reserved to glutamic acids (E), either individually or in clusters, thus generating single, double, and triple D_2L_-R mutants. Through analyses of cellular localization by confocal microscopy and enzyme-linked immunosorbent assay (ELISA), radioligand binding assay, bioluminescence resonance energy transfer (BRET^2^) β-arrestin 2 (βarr2) recruitment assay, and cAMP signaling, it was revealed that charge reversal of the R residues at all three positions within the motif impaired their colocalization with ER marker calnexin and led to significantly improved cell surface expression. Additionally, these data demonstrate that an R to glutamic acid (E) substitution at position 2 within the RXR motif is not functionally permissible. Furthermore, all generated D_2L_-R mutants preserved their functional integrity regarding ligand binding, agonist-induced βarr2 recruitment and Gα_i_-mediated signaling. In summary, our results show that the conserved arginine cluster within the 29-amino acid insert of third cytoplasmic loop (IC3) of the D_2L_-R appears to be the ER retention signal.

## 1. Introduction

The effects of the neurotransmitter dopamine are mediated by several types of dopamine receptors, which are G-protein-coupled receptors (GPCRs). Five types of dopamine receptors have been identified, and based on their structural, functional, and pharmacological properties, they are categorized as either D1 or D2 receptors, which stimulate and inhibit adenylyl cyclase (AC) activity, respectively. D2-like receptors (D_2_, D_3_, and D_4_) have long third cytoplasmic loops and short carboxyl-terminal tails and include the receptor variants generated by alternative splicing (D_2_ and D_3_) or polymorphic variation (D_4_) (reviewed by Beaulieu et al. [1]). Two alternatively-spliced variants of the D_2_ receptor were classified as short (D_2S_-R) and long (D_2L_-R) receptor isoforms. The presence of the 29-amino acid insert in the third cytoplasmic loop (IC3) of the D_2L_-R [2,3,4] has led to speculation that this insert might determine the functional differences between D_2S_-R and D_2L_-R in regards to G protein coupling [5,6,7], post-translational modification [8], and in vivo function, as D_2S_-R and D_2L_-R participate in presynaptic and postsynaptic dopaminergic transmission, respectively [9,10]. Data obtained with the D_2_-R knockout mice provided additional evidence for their different roles in motor and cognitive functions [11], responsiveness to cocaine [12], and therapeutic/side effects of antipsychotic agents [13]. D_2_-R isoforms also displayed differences in their intracellular localization and trafficking. Studies reported predominant intracellular localization of the D_2L_-R in the primate brain [14] and different cell lines [15,16,17], whereas D_2S_-R displayed predominant plasma membrane (PM) localization [16,17]. The primary intracellular localization site of the D_2L_-R in transiently transfected COS-7, HeLa, and HEK-293 cells was the endoplasmic reticulum (ER) [15], whereas retention in the perinuclear region and Golgi apparatus (GA) was observed in transfected NG108-15 cells [17]. Ligand-promoted recruitment of the D_2L_-R [18], D_4_-R [19], and other GPCRs, i.e., thrombin receptors (protease-activated receptor 1 (PAR1) and PAR2), D_1_-Rs, and δ-opioid receptors, to the PM provided further evidence for the existence of pre-existing, functional intracellular receptor stores (reviewed in [20,21]).

**Figure 1:**
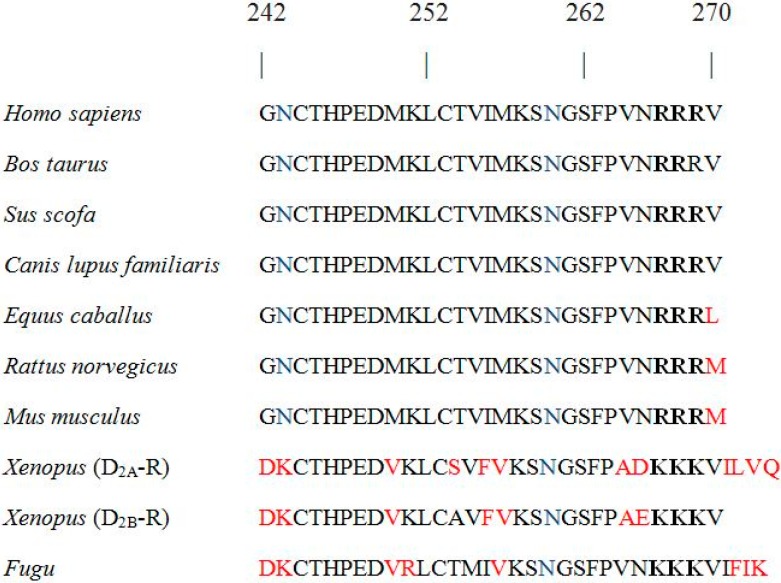
Sequence alignment of the alternatively-spliced exon in the long form of the D_2_ dopamine receptor (D_2L_-R). Comparisons of the amino acid sequence show its high conservation among species. Amino acids that differ from the sequence of the insert in humans are indicated in red. Potential *N*-glycosylation sites (N243 and N260) are marked in blue. The conserved sequence of three basic amino acids (arginine (R) or lysine (K)), which represent a potential endoplasmic reticulum (ER) retention motif, is written in bold.

GPCR export from the ER and GA and their targeting to the PM is regulated by several factors, including (i) conserved motifs necessary for receptor export from the ER and GA; (ii) folding in the ER; (iii) ER-localized chaperone proteins; (iv) interacting proteins modulating anterograde transport; (v) homo-/heteromerization (reviewed by Dong et al. [22] and Milligan [23]); and (vi) chemical and pharmacological chaperones (reviewed by Babcock and Li [24]). On the other hand, retention in the ER could be due to the presence of specific ER retention signals [22] and their postulated interactions with the ER-resident gatekeeper proteins [25]. Three types of ER retention motifs were identified in the intracellular regions of various proteins, i.e., KDEL, KKXX, and RXR amino acid sequences, where K is for lysine, D/E is for aspartic/glutamic acid, L is for leucine, R is for arginine and X is for any amino acid. KDEL and KKXX are supposed to act as retrieval signals that recycle proteins from the GA back to the ER, whereas the RXR motif prevents their exit from the ER [22]. In the case of the prototypic γ-aminobutyric acid B1 receptor (GABA_B1_), the arginine-based RXR was identified in the C-terminal tail [26]. It was subsequently demonstrated that the RXR motif and the upstream di-leucine (LL) export motif [27] are both engaged in the interaction with the prenylated Rab acceptor family 2 (PRAF-2), thus preventing its PM trafficking in the absence of hetero-dimerization with GB2 [28]. RXR-type ER retention signals were also described within the carboxyl terminals of the Kir6.2 potassium channel [29], α-adrenergic receptor-type 2c (α_2c_-AR) [30], G-protein coupled receptor 15 (GPR15) [31], and in the intracellular loop of the kainate receptor subunit KA2 [32]. Considering that the only difference between the D_2_-R isoforms is the presence of an additional 29 amino acids in the IC3 of D_2L_-R, we hypothesized that the molecular determinants underlying its pronounced retention in the ER/intracellular compartment could be present within this insert. Comparisons of the alternatively-spliced exon amino acid sequences (Genbank database) showed high evolutionary conservation among species (Figure 1). There are two potential glycosylation sites in the 29-amino acid insert (N243 and N260), although their physiological importance is presently unknown. In the terminal end of the insert is a set of basic arginine residues (R267, R268 and R269, mammals), which could represent a conserved RXR-type ER retention motif (Figure 1).

To test our hypothesis, we mutated conserved basic arginine residues in the presumed ER retention motif into glutamic acid (E) and assessed the cellular localization and functional properties of D_2L_-R and its mutants using confocal microscopy, enzyme-linked immunosorbent assay (ELISA), radioligand binding assay, BRET^2^ β-arrestin 2 (βarr2) recruitment assay, and cAMP signaling. Our data suggest that the conserved arginine cluster in the insert of IC3 of the D_2L_-R acts as an ER retention signal.

## 2. Results

### 2.1. Characteristics of the Generated Long Form of the D_2_ Dopamine Receptor (D_2L_-R) Mutants

D_2L_-R and generated mutants were first pharmacologically characterized by radioligand binding assay using the selective, hydrophilic D2-Rs antagonist sulpiride, which only binds to membrane-localized receptors. In HEK-293 cells expressing D_2L_-R, sulpiride competed for binding against the [^125^I]-iodosulpride with an affinity of 0.82 ± 0.17 nM. This value is in good agreement with those reported for rat and rabbit striatal membranes [33] and human D_2_-Rs expressed in CHO cells [34]. The IC_50_ values obtained with the generated mutants did not significantly differ from those obtained for the D_2L_-R (*p* = 0.281) (Table 1). Data from the radioligand self-displacement assays were also used to assess surface receptor density (B_max_) (Table 1). All D_2L_-R mutants showed a trend toward an increased surface receptor density, but due to high inter-assay variations in their surface expression, this did not reach statistical significance (*p* = 0.587) (Table 1).

### 2.2. Visualization and Cellular Localization of the D_2L_-Rs—Colocalization Study

**Figure 2:**
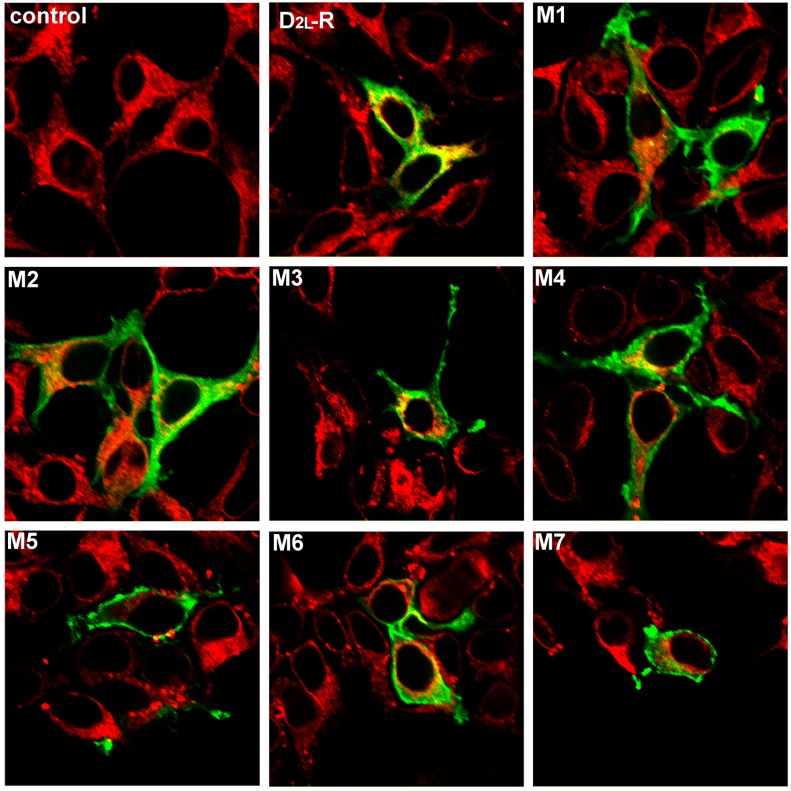
Visualization of the D_2L_-R and D_2L_-R mutants and their cellular localization and colocalization with calnexin in transiently transfected HEK-293 cells. D_2L_-R and D_2L_-R mutant (M1–M7) cellular localization was assessed by confocal microscopy in unstimulated cells. The green color indicates D_2L_-R and D_2L_-R mutants; red indicates calnexin, an ER marker; yellow/orange indicates colocalization of an individual D_2L_-R construct and calnexin. Note that all D_2L_-R mutants displayed reduced colocalization with calnexin and more apparent localization to the cell surface compared to the D_2L_-R. Objective 40× and zoom factor 4 apply for all images.

The cellular distributions of the D_2L_-R and generated mutants were examined by indirect immunofluorescent staining. Figure 2 shows the distribution of the D_2L_-R and the mutants thereof at a steady state (untreated cells; green signal) and their colocalization with the ER marker calnexin (red signal). Confocal fluorescence microscopy demonstrated a substantial proportion of the D_2L_-Rs in the intracellular compartment largely overlapped with calnexin (yellow/orange signal). The fluorescent signal obtained with the D_2L_-R mutants was confined mainly to the cell surface. In addition, a small intracellular receptor pool was detected with M2, M3, and M4 that partially co-localized with calnexin. The degree of colocalization between the D_2L_-R constructs and calnexin was quantitatively assessed by calculating Pearson’s correlation coefficient for images above thresholds (Rcoloc; Supplementary Material, Table S1). D_2L_-R and calnexin showed a high degree of colocalization (Rcoloc ~0.76), while lower Rcoloc (0.40 to 0.51) obtained for the D_2L_-R mutants suggest a substantial decrease of colocalization.

### 2.3. D_2L_-R Constructs Surface Expression—Enzyme-Linked Immunosorbent Assay (ELISA)

**Figure 3:**
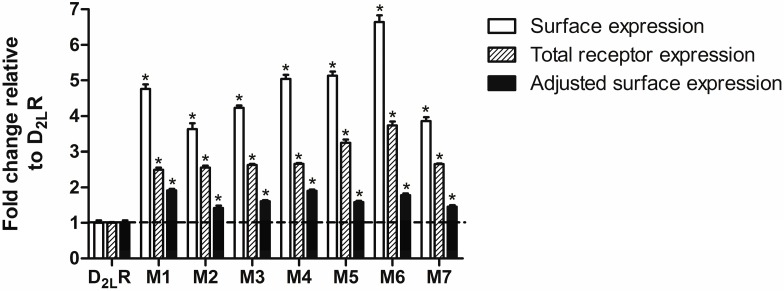
Expression levels of D_2L_-R and D_2L_-R mutants in transiently transfected HEK-293 cells. Intact HEK-293 cells transiently transfected with D_2L_-R/RLuc8 or individual D_2L_-R/RLuc8 mutants were used in an Enzyme-Linked Immunosorbent Assay (ELISA). Receptor surface expression was assessed by measuring the absorbance at 450 nm; the background values obtained with untransfected HEK-293 were subtracted from all readings (open bars). The D_2L_-R/RLuc8 construct total expression i.e., surface and intracellular were determined by luminescence measurements as described in the Materials and Methods section (hatched bars). Closed bars represent adjusted surface expression normalized against the total receptor expression. Data are expressed as the mean ± standard error of the mean (S.E.M.) of three independent experiments performed in triplicate. Significance relative to the D_2L_-R was determined by one-way ANOVA with Dunnett’s post hoc test (*, *p* < 0.05).

The effect of mutated arginine residues in the presumed RXR-type ER retention motif on D_2L_-R surface expression was then quantitatively assessed by ELISA. C-terminally RLuc8-tagged D_2L_-R constructs were used in ELISA and all subsequent experiments, as they allowed us to monitor the total (surface and intracellular) receptor expression and perform data adjustment to the total receptor expression. As shown in Figure 3, a significant and approximate four- to six-fold increase in the surface expression was observed in HEK-293 cells expressing D_2L_-R mutants compared with cells expressing D_2L_-R (open bars). Total luminescence measurements revealed that the expression levels of individual mutants were significantly higher than that of D_2L_-R (hatched bars), which could underlie the increased surface expression of the mutant forms of the D_2L_-R. The increases in the surface expression of D_2L_-R/RLuc8 mutants determined by ELISA were then adjusted by total expression levels using the RLuc8 signal quantified by total luminescence measurements. After adjustment of the surface expression data to the total receptor expression, the surface expression of individual mutants was lower, but still significantly higher (up to ~2-fold) compared to D_2L_-R (closed bars). To achieve comparable total expression levels, HEK-293 cells were also transfected with different amounts of cDNA encoding either D_2L_-R/Rluc8 or M1/RLuc8. In the case that comparable total expression of D_2L_-R/Rluc8 and M1/RLuc8 was achieved, i.e., 1.00 ± 0.01 and 0.89 ± 0.03, surface expression of M1/RLuc8 was significantly higher (1.00 ± 0.08 vs. 2.73 ± 0.14). The experimental M1/RLuc8 surface expression level was slightly higher, but it was still in a range comparable with that for the M1/RLuc8 adjusted surface expression (1.91 ± 0.05), thus providing the rationale for the total receptor expression adjustments.

### 2.4. D_2L_-R Constructs Functional Characterization—Bioluminescence Resonance Energy Transfer (BRET^2^) and cAMP Assay

**Figure 4:**
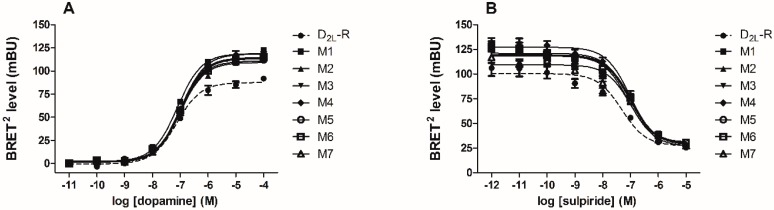
BRET^2^-based agonist and antagonist dose-response curves for the D_2L_-R and D_2L_-R mutants. HEK-293 cells were transfected transiently with the indicated RLuc8-tagged D_2L_-R construct and GFP^2^/βarr2 R393E,R395E at a 1:14 cDNA ratio. For the agonist dose-response curves (**A**); increasing concentrations (10^−11^ to 10^−4^ M) of dopamine were added to the cells. For antagonist dose-response curves (**B**); cells were first treated with increasing concentrations (10^−12^ to 10^−5^ M) of the antagonist sulpiride for 15 min. Subsequently, dopamine was added, resulting in a final concentration of 1 μM. BRET^2^ signals were measured as described in the Materials and Methods. The data are expressed as the mean ± standard deviation (S.D.) of triplicate observations from a single experiment and are representative of at least three measures.

BRET^2^ assay was used to monitor the recruitment of green fluorescent protein 2 (GFP^2^)/βarr2 constructs to RLuc8-tagged D_2L_-R and D_2L_-R mutants. A more than two-fold increase in the maximal agonist-induced BRET signal (BRET_max_) was obtained with the double GFP^2^/βarr2 R393E,R395E mutant compared to GFP^2^/βarr2, whereas the potency of dopamine was comparable, i.e., 262.8 ± 85 and 140.5 ± 31.6 nM for the GFP^2^/βarr2 and GFP^2^/βarr2 R393E,R395E, respectively. This is in agreement with our previous observations for other GPCRs [35,36] and also for the D_2S_-R [37]. The later study also reported that both D_2S_-R and D_2L_-R displayed comparable potencies in the BRET βarr2 recruitment assay. Due to a larger signal window, the GFP^2^/βarr2 R393E,R395E mutant was used in subsequent experiments. The constitutive BRET^2^ signal (BRET_const_) generated by non-activated receptor interaction with the GFP^2^/βarr2 R393E,R395E mutant was comparable between D_2L_-R and individual mutants. Stimulation of the D_2L_-R/RLuc8 and D_2L_-R/RLuc8 mutants by dopamine produced a dose-dependent increase in the BRET signal (Figure 4A). BRET^2^ antagonist dose-response curves generated in the presence of increasing concentrations of the D_2L_-R antagonist sulpiride are shown in Figure 4B. The agonist-induced BRET_max_ signal and the half-maximal effective concentrations (EC_50_) and the half maximal inhibitory concentration (IC_50_) values from the BRET^2^ agonist and antagonist dose-response curves for wild type (WT) and D_2L_-R mutants are summarized in Table 2. The BRET_max_ obtained with M1–M6 was significantly higher than the agonist-induced BRET_max_ signal generated from D_2L_-R interactions with the βarr2 R393E,R395E, whereas there were no substantial changes in the tested ligand potencies.

**Figure 5:**
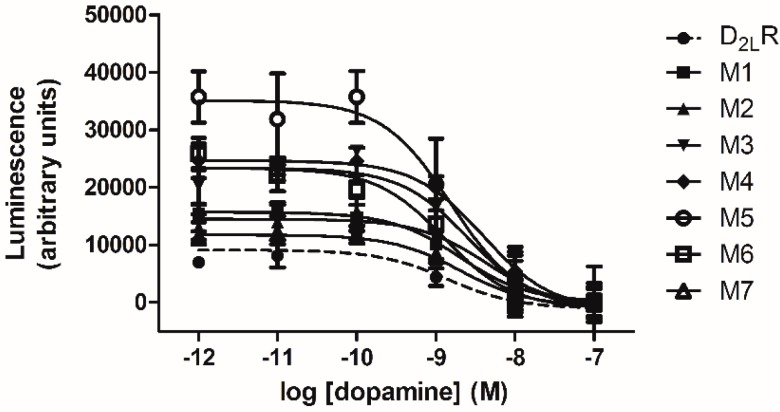
Inhibition of forskolin-stimulated cAMP accumulation by agonist-activated D_2L_-R and D_2L_-R mutants. Decreases in cAMP through D_2L_-R construct-induced G_i_ activation were determined in HEK-293 cells transiently transfected with D_2L_-R/RLuc8 or individual D_2L_-R/RLuc8 mutants (M1/Rluc8-M7/RLuc8). Shown in the figure are the dose-response curves of dopamine for inhibiting cAMP accumulation induced by 20 μM of forskolin in HEK-293 cells. Total expression of D_2L_-R/RLuc8 constructs was also determined by luminescence measurement as described in the Materials and Methods. A dopamine-induced signal reduction was calculated by subtracting the background signal (signal not displaced by the highest 10^−7^ M concentration of dopamine) from the maximal signal measured in the absence of agonist and then adjusting for the total receptor expression. The data shown are the mean ± standard deviation (S.D.) of quadruple observations from a single experiment representative of at least three.

As G_i/o_ protein-mediated cAMP inhibition is the best response for characterizing D2-Rs activation, the ability of D_2L_-R and individual D_2L_-R mutants to inhibit forskolin-mediated activation of adenylyl cyclase was also measured to assess their capacity to initiate signaling via G_i/o_ proteins. As shown in Figure 5, dopamine inhibited forskolin-stimulated cAMP accumulation in live HEK-293 cells expressing D_2L_-R or individual D_2L_-R mutants. Low concentrations of dopamine (up to 10^−7^ M) were used to generate dose-response curves, as it was previously shown that cAMP increases at higher concentrations due to endogenously-expressed D1-Rs in HEK-293 cells [38]. The maximal agonist-induced decrease in cAMP accumulation and the calculated EC_50_ values derived from the agonist dose-response curves for the WT and D_2L_-R mutants are summarized in Table 3. There were no substantial differences in the potency of dopamine to inhibit forskolin-stimulated cAMP accumulation, whereas the efficacy of M3, M4, M5, and M6 was significantly higher than that of the D_2L_-R.

## 3. Discussion

This study was focused on elucidating the mechanisms underlying predominant intracellular localization of the D_2L_-R that has been reported in different heterologous expression systems and native tissues [14,15,16,17] and could underlay differential ability of both isoforms to increase steady-state receptor concentration induced by prolonged agonist/antagonist treatment in different transfected cell lines [18,39,40,41]. Similarly, D_4_-R repeat variants also displayed differences in pharmacological chaperone-mediated upregulation, thus suggesting that the ligands may stabilize a specific receptor conformation and so improve their processing through the ER [19]. A regulated pool of intracellular receptors could also provide a mechanistic basis for behavioral supersensitivity to D2 agonists reported in animals exposed to indirect dopamine agonists [42,43] and for a sustained clinical efficiency of dopamine agonists during long term therapy in patients (reviewed in [21]). We hypothesized that this is an intrinsic property of the D_2L_-R and that the conserved arginine cluster located within the 29-amino acid insert could act as an intracellular retention signal. A triple arginine cluster (R367–R369) represents the prospective RXR-type ER retention motif, which, unlike the C-terminal–K(X)KXX ER-localization signal, is present in different cytosolic domains of integral membrane proteins [22,44]. The cluster position within the IC3, which is a certain distance from the PM, is also suggestive of its functionality, as many proteins that have the RXR motif near the cytoplasmic side of the PM are not retained in the ER [45,46]. Supporting the latter, a further set of four arginine residues (R217–R220) located toward the N-terminal region of IC3 close to the TM5 of D_2_-Rs was shown to be essential for efficient PM localization and heteromerization with the D_1_-R [47] and other GPCRs, including adenosine A_2A_ receptor [48]. However, subsequent studies revealed that arginine residues adjacent to the insert (R374 and R375) are involved in forming heteromers with the D_1_-R [49] and D_5_-R [50]. Even though that arginine residues common to both D_2_-R isoforms were implicated as the possible heteromer interacting site, protein generated from the 29-amino acid insert of D_2L_-R disrupted the D_1_–D_2_ receptor heteromer and showed antidepressant-like effects in mice (reviewed in [51]).

It was initially observed that D_2L_-R mutants with charge-reversed basic R residues within the prospective ER retention motif showed increased cell surface expression and reduced colocalization with the ER marker calnexin, whereas their ligand-binding properties were not significantly affected. The study by Guiramand et al. [52], which also evaluated the importance of certain amino acids residues within the IC3 of the D_2L_-R, revealed that mutants with basic residues substituted with nonpolar valine within (K251, K258, R367, R368, and R369), and adjacent to, the insert (R374 and R375) displayed an unchanged or a slightly higher binding affinity for spiperone, but their expression was decreased. This would appear to contradict our observations, but it should be noted that a single mutation, i.e., K251V, already reduced the B_max_ by more than 30% and that the mutants in which only R residues were in or adjacent to the insert were not generated. Additionally, mutation of a single lysine residue in the IC1 of α_2A_-adrenergic receptor (AR) (K65A) positioned next to leucine (L64) decreased the receptor cell surface expression [53]*.* Hurt et al. [54] proposed that identification of the potential ER motif requires special attention in regards to the membrane/total receptor expression ratio and the mutant functional properties. Therefore, we employed a previously described approach [55] and used double N-terminally HA- and C-terminally RLuc8-tagged receptor constructs that allowed us to monitor the surface and total receptor expression. ELISA and total luminescence measurements showed higher surface and total expression of all generated mutants. However, their adjusted surface expression normalized against the total receptor expression was significantly higher than that for the D_2L_-R. Together with the data obtained from the confocal colocalization study, these data suggest that mutation of the potential RXR-type ER retention motif interfere with the ability of D_2L_-R to maintain substantial intracellular receptor pool. The strength of the ER retention motif depends on the number of R residues, whereas the residue preceding RXR and the characteristics of the amino acids at position X modulates its efficacy (reviewed in [44]). The data obtained with single mutants showed that the charge reversal of either R residue rendered the motif inactive, with only a small difference in their efficacy. Additionally, no substantial additive effect on the receptor surface expression was observed with either double or triple mutants. The effect obtained with the single R368E (M2; RER) corroborates the observation that negatively charged residues at position X makes the motif inactive [56]. Functional characterization of the D_2L_-R and generated mutants was done by BRET-based βarr2 recruitment assay and cAMP signaling. All mutants showed some differences in their efficacies, but not in their tested ligand potencies. However, it should be emphasized that only PM-localized receptors were stimulated, as dopamine is membrane-impermeable. βarr2 recruitment assay was used as the IC3, which is the main site of interaction with β-arrestins for many GPCRs, including D_2L_-R [57], and the suitability of this assay for the D_2_-Rs was confirmed in previous studies [37,38,58]. The applicability of our previously described βarr2 R393E,R395 mutant, which shows an overall two-fold improvement in the BRET ratio [35], had also been demonstrated for D_2S_-Rs [37], which, similar to D_2L_-R, showed only small or no changes in the compound potency. Most of the tested ligands also displayed comparable efficacies and potencies in recruiting βarr2 to either D_2S_-R or D_2L_-R [37]. Moderate constitutive interactions of the D_2L_-R constructs with the βarr2 R393E,R395 are in agreement with a previous report obtained with the murine D_2L_-R/βarr2 pair expressed in HEK-293 cells [38]. The maximal agonist-induced BRET^2^ signal (BRET_max_) obtained for the D_2L_-R mutant interaction with the βarr2 R393E,R395 was up to 1.5-fold higher than that for the D_2L_-R. This could be due to small changes in the orientation of the donor and acceptor molecules and/or fluctuations in their relative expression levels [59]. Most importantly, the D_2L_-R mutants displayed unchanged potency when tested in agonist and antagonist modes. This argues against the involvement of the arginine cluster within the IC3 in the interaction with βarr2. Actually, four residues at the N-terminal region of IC3 are common to both D_2_-R isoforms, i.e., IYIV212 comprise the βarr2 binding domain [60]. The cAMP data also provided evidence for the preserved functional integrity of D_2L_-R mutants. Similarly, substitution of the 29-amino acid insert with an equivalent length epitope had no functional consequences in regards to cAMP signaling [61]. The improved efficacy correlates with their higher surface expression, whereas the potency of dopamine in inhibiting forskolin-mediated activation of AC was comparable with that for the WT D_2L_-R and those previously reported for the rat D_2L_-R expressed in HEK-293 cells [60]. It was previously reported that the mutants S259A, S262A, and D249V located within the insert displayed increased potency in inhibiting AC, whereas this was not the case when basic residues were mutated [52]. The BRET and cAMP data, therefore, provide evidence that the D_2L_-R mutants have preserved functional properties in regards to Gα_i_-coupling and βarr2 recruitment, thus further pointing to the role of a conserved arginine cluster (R367–R369) as an ER retention motif.

Exit from the ER is a highly-regulated process critical for the anterograde transport of GPCRs through the GA to the PM and their surface expression [22]. Therefore, it is unlikely that only the RXR-type ER retention motif would be involved. Over the years, several underlying mechanisms have been proposed for the D_2L_-R intracellular retention, which have included (i) partial glycosylation in the post-ER compartment [8]; (ii) constitutive, clathrin- and dynamin-independent endocytosis of the D_2L_-R [62]; (iii) interaction with the intracellularly-located Gα_i2_ splice variant [63]; and (iv) interaction with a specific ER-resident gatekeeper protein, such as GTPase 6 interacting protein 5 (ARL6IP5) (previously named as GTRAP3-18, Yip6b or PRAF-3) [64]. The RXR motif is probably involved in the interaction with the ER-resident gatekeeper protein, and PRAF-3 could be a candidate interaction partner, as it has been shown to confine β_2_-AR and D_2_-R to the ER [64]. Such a role has recently been reported for the related ER-resident gatekeeper PRAF-2 in GABA_B_ receptor cell surface export [28]. Studies have attempted to identify specific interacting partners for the IC3 of D_2L_-R, but were largely unsuccessful because most of the identified partners including Gα_i2_ splice variant bind to the IC3 of both isoforms [65,66]. So far, only one protein, i.e., heart-type fatty acid binding protein (H-FABP), has been identified that specifically interacts with the 29-amino acid insert region of D_2L_-R [17]. However, due to its involvement in the regulation of D_2_-R functions [67], it is unlikely to be involved in its anterograde trafficking to the PM. Additionally, a naturally-occurring synonymous mutation of human D_2_-R (C957T, Pro319Pro) postulated to correlate with the schizophrenia phenotype was shown to markedly change mRNA stability and reduce dopamine-induced upregulation of D_2_-R expression [68].

Taken together, the present study presents evidence that the evolutionary conserved arginine cluster located within the 29-amino acid insert could act as an ER motif and be a part of the intricate underlying mechanisms responsible for differentially-regulated anterograde trafficking of the D_2_-R isoforms and their PM availability.

## 4. Materials and Methods

### 4.1. Materials

Molecular biology reagents, tissue culture reagents, and media were from Sigma-Aldrich (St. Louis, MO, USA) and Gibco Invitrogen Corporation (Breda, The Netherlands). [^125^I]-iodosulpride was purchased from PerkinElmer (Boston, MA, USA). Dopamine and (*S*)-(−)-sulpiride were obtained from Sigma-Aldrich and coelenterazine 400a from Biotrend Chemikalien GmbH (Köln, Germany). Anti-hemagglutinin (HA) rat monoclonal and anti-calnexin rabbit polyclonal antibodies were from Roche (Basel, Switzerland) and Sigma-Aldrich, respectively. Anti-rat horseradish peroxidase (HRP)-conjugated, anti-rat fluorescein isothiocyanate (FITC)-conjugated and anti-rabbit tetramethylrhodamine (TRITC)-conjugated antibodies were purchased from Sigma-Aldrich.

### 4.2. Receptor and β-Arrestin 2 Constructs

**Figure 6:**
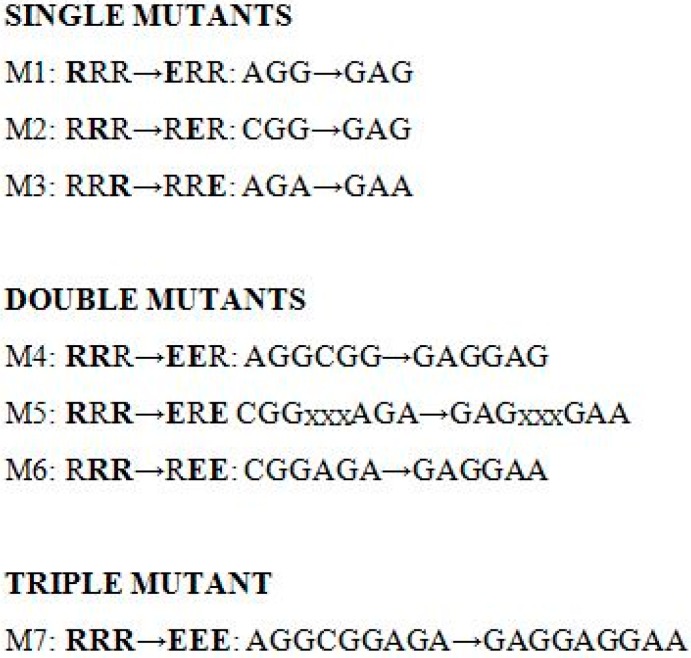
D_2L_-R mutants used in the study. Basic arginine (R) residues at positions 267, 268, and 269 in the insert of the D_2L_-R and D_2L_-R/RLuc8 were charge-reversed into glutamic acid (E), either individually or in clusters, to generate single, double, and triple mutants. Mutated residues are written in bold. “xxx”, unchanged nucleotide triplet.

Human D_2L_-R N-terminally-tagged with triple HA-tag in the vector pcDNA3.1(+) was obtained from the Missouri S and T cDNA Resource Center (University of Missouri-Rolla, Rolla, MO, USA) and is hereafter referred to as D_2L_-R. D_2L_-R C-terminally tagged with *Renilla luciferase* 8 (RLuc8) (D_2L_-R/RLuc8) was generated according to standard molecular biology techniques and verified by sequencing. Arginine (R) residues in the D_2L_-R and D_2L_-R/RLuc8 at positions 267, 268, and 269 were mutated to E, either individually or in clusters, thus generating single, double, and triple mutants (Figure 6) that are hereafter referred to as M1–M7 and M1/RLuc8–M7/RLuc8. The mutants were generated and verified by sequencing at Genscript USA Inc. (Piscataway, NJ, USA). Human β-arrestin 2 (βarr2) N-terminally tagged with GFP^2^ (GFP^2^/βarr2) (PerkinElmer BioSignal, Inc. (Montreal, ON, Canada)) was cloned into the vector pcDNA3.1(+) using the NheI/XbaI restriction sites. The GFP^2^/βarr2 R393E,R395E mutant was the same as that described previously [35]. Double GFP^2^/βarr2 R393E,R395E mutant is phosphorylation-independent and defective in their ability to interact with the components of the clathrin-coated vesicles, thus yielding an augmented and more stable BRET^2^ signal.

### 4.3. Cell Culture and Transfection

Human embryonic kidney (HEK)-293 cells (European Collection of Animal Cell Cultures (Salisbury, UK)) were routinely cultured in Dulbecco’s modified Eagle’s medium (DMEM) with 10% (*v*/*v*) heat-inactivated fetal bovine serum (FBS), 2 mM Glutamax-I, penicillin (100 U/mL), and streptomycin (100 μg/mL) at 37 °C in a humidified atmosphere of 5% (*v*/*v*) CO_2_. Transient transfections were performed following the Lipofectamine^®^-*Plus*™ *Reagent* protocol when cells reached ~90% confluence.

### 4.4. Confocal Microscopyand Quantification of Co-Localization

The cellular distribution of D_2L_-R constructs in transiently-transfected HEK-293 and their possible co-localization with the ER marker calnexin was assessed by confocal microscopy using a previously-described protocol [69,70]. Briefly, HEK-293 cells transiently transfected with 1 μg of individual D_2L_-R constructs were grown on poly-d-lysine-coated glass coverslips. After 48 h, cells were fixed with 4% paraformaldehyde and then washed with phosphate*-*buffered saline (PBS), permeabilized with 0.01% Triton X-100 in PBS, then incubated in a blocking solution (1% bovine serum albumin (BSA) in PBS) to reduce the nonspecific binding. Next, cells were incubated overnight at 4 °C with a 1:100 dilution of rat anti-HA and 1:100 dilution of rabbit anti-calnexin antibodies. After extensive washing, cells were incubated for 60 min at room temperature in the dark with a 1:50 dilution of secondary anti-rat FITC- and anti-rabbit TRITC-conjugated antibodies. After washing, cells were mounted using an anti-fading ProLong^®^Gold reagent (Molecular Probes, Leiden, The Netherlands), sealed and examined under an oil immersion objective (Planapo 40×, numerical aperture (N.A.) = 1.25) using a Leica multispectral confocal laser microscope (Leica TCS NT, Heidelberg, Germany). Sequential images were collected in an eight-fold frame with an average resolution of 1024 × 1024 pixels. Representative sections corresponding to the middle of the cells are presented using Adobe Photoshop 7.0. 

The degree of co-localization was quantified using ImageJ [71] and the Colocalization plugin [72] image analysis software. Pearson’s correlation coefficient for image above thresholds (Rcoloc) was calculated, which represents pixels where both channels (red and green) were above their respective threshold; its values range between −1.0 and 1.0, where −1, 0, and +1 indicate complete negative correlation, no significant correlation and perfect correlation, respectively. Five to seven individual cells showing co-localization between the individual D_2L_-R construct and calnexin were analyzed.

### 4.5. Radiolig and Binding Assay

Self-displacement radioligand binding assay performed on whole cells was based on a previously-described protocol [35,69,70]. The hydrophilic, membrane-impermeable ligand [^125^I]-iodosulpride was used to determine the number and binding properties of cell surface-expressed D_2L_-R and individual D_2L_-R mutants (M1–M7). After transfection with 4 μg of cDNA (75 cm^2^ flask) for D_2L_-R or individual D_2L_-R mutants (M1–M7), cells were plated onto 24-well plates at a density of ~1 × 10^5^ cells per well, and the assay was performed after 48 h. Cells were incubated with the [^125^I]-iodosulpride (30.000 cpm/well) and increasing concentrations (10^−12^ to 10^−5^ M) of unlabeled -/-sulpiride in assay buffer (HEPES-modified DMEM with 0.1% BSA) for 30 min at 25 °C. After washing in ice-cold PBS, cells were solubilized with 0.2 M NaOH and 1% sodium dodecyl sulfate (SDS) solution and the radioactivity levels determined using a γ counter (LKB Wallac, Turku, Finland). All treatments were performed in triplicate in at least three independent experiments. The binding parameters were obtained from self-displacement curves (GraphPad Prism 5.0) and the receptor density (B_max_) calculated as previously described by Ramsay et al. [73].

### 4.6. Luminescence and Fluorescence Measurements

Total luminescence and fluorescence measurements were performed as previously described [36] to deterimine the expression levels of RLuc8-tagged D_2L_-R receptor and GFP^2^-tagged β-arrestin 2 constructs. Signals were measured in a TriStar^2^ LB 942 microplate reader (Berthold Technologies, Bad Wildbad, Germany). Background values obtained with mock-transfected HEK-293 cells were subtracted in both measurements, and the mean values of triplicate wells/sample were calculated.

### 4.7. Enzyme-Linked Immunosorbent Assay (ELISA)

ELISA was used to establish the level of surface-expressed RLuc8-tagged D_2L_-R constructs, while the luminescence was measured to determine total receptor expression. ELISA was performed as previously described [69,70]. HEK-293 cells were transiently transfected with 1 μg of cDNA for D_2L_-R/RLuc8 or individual D_2L_-R/RLuc8 mutants (M1/RLuc8–M7/RLuc8). To achieve comparable expression levels, different amounts of cDNA were also used, ranging from 0.5 to 2.5 μg, and the total amount of cDNA was kept uniform by adding an empty pcDNA3.1 vector. After transfection, the cells were seeded in a 24-well plate (~1 × 10^5^ cells/well). ~3 × 10^5^ cells from each transfection were also seeded into a 60-mm dish for total luminescence measurements. After 48 h, cells were first serum-starved for 2-h and then fixed with 4% paraformaldehyde, washed in PBS, and blocked (1% BSA in PBS). Subsequently, cells were incubated at 4 °C overnight with a 1:600 dilution of anti-HA antibody. After three washes, cells were incubated with anti-rat HRP-conjugated antibody at a 1:1000 dilution and, finally, the 3,3′,5,5′-tetra-methylbenzidine (TMB) liquid substrate system (Sigma) was used to develop the reaction, which was stopped after 10 min at 37 °C by adding 0.2 N sulfuric acid solution. The absorbance was measured at 450 nm in a Rosys Anthos 2010 microplate reader (Anthos Labtec Instruments, Wals, Austria). HEK-293 cells transfected with empty vector pcDNA3.1 were used to determine the background, which was subtracted from all readings. Determinations were made in triplicate.

### 4.8. BRET-Based β-Arrestin 2 Recruitments Assay

The ability of agonist-activated D_2L_-R constructs to recruit βarr2 was determined utilizing a previously-described BRET-basedβarr2 recruitments assay [35,36]. Briefly, HEK-293 cells in a 75 cm^2^ flask were transiently co-transfected with constructs encoding individual D_2L_-R/RLuc8 constructs (0.4 μg) together with constructs encoding GFP^2^-βarr2 (5.6 μg) or double GFP^2^-βarr2 R393E,R395E mutant (5.6 μg). At 48 h after transfection, HEK-293 cells were trypsinized, washed once in Dulbecco’s phosphate-buffered saline (DPBS), and resuspended in DPBS supplemented with Ca^2+^/Mg^2+^, 1 g/L glucose, and 36 mg/L sodium pyruvate. Resuspended cells (~2 × 10^5^) were distributed in 96-well microplates (white Optiplate; Packard BioScience, Meriden, CT, USA) and treated with increasing concentrations (10^−4^ to 10^−11^ M) of dopamine and incubated for 5 min. For the antagonist assay, the antagonist sulpiride (10^−5^ to 10^−12^ M, final concentration) was added to the cells 10 min prior to the addition of the dopamine (1 μM, final concentration). The substrate coelenterazine 400a was then injected with a final concentration of 5 μM. The signals detected at 395 and 515 nm were measured sequentially, and the 515/395 ratios were calculated and expressed as a miliBRET level (mBU; BRET ratio × 1000). Expression levels of RLuc8- and GFP^2^-tagged constructs for each experiment were determined by total luminescence and fluorescence measurement.

### 4.9. cAMP Assay

The HitHunter^®^ cAMP assay (DiscoveRx Corporation Ltd.) was used to determine the ability of D_2L_-R constructs to inhibit forskolin-stimulated cAMP accumulation and was performed according to the manufacturer’s instructions. HEK-293 cells in 60 mm dishes were transiently transfected with 1 μg of cDNA encoding individual D_2L_-R constructs. After 48 h, cells were trypsinized, resuspended in a cell assay buffer (PBS with 0.5 mM 3-isobutyl-1-methylxanthine (IBMX)), and seeded (5 × 10^4^ cells/well) onto 96-well plates (white, Optiplate). Cells were then treated for 60 min at room temperature with increasing concentrations (10^−13^ to 10^−7^ M) of dopamine diluted in PBS in the presence of forskolin (20 μM, final concentration). HitHunter cAMP reagents were then added sequentially, and the cells were incubated in the dark at room temperature. Total luminescence was read after 6 h in a TriStar LB 942 microplate reader at 1 s/well and data analysis performed using GraphPad Prism 5.0. Expression levels of RLuc8-tagged constructs in each experiment were monitored by total luminescence measurement.

### 4.10. Statistical Analysis

The results are expressed as the mean ± S.E.M. The statistical significance relative to the D_2L_-R (control) was determined by one-way ANOVA, followed by Dunnett’s post hoc test. Statistical analyses were conducted by SPSS 20.0 for Windows (SPSS Inc., Chicago, IL, USA). A *p*-value ≤0.05 was regarded as statistically significant.

## Figures and Tables

**Table 1:** The pharmacological properties and D_2L_-R and D_2L_-R mutant surface receptor density (B_max_) in transiently transfected HEK-293 cells. HEK-293 cells transiently transfected with D_2L_-R or individual D_2L_-R mutants were incubated with [^125^I]-iodosulpride and increasing concentrations (10^−12^ to 10^−5^ M) of sulpiride. The IC_50_ values were obtained by sigmoidal dose-response curve fit (GraphPad Prism 5.0, GraphPad Software, San Diego, CA, USA) and B_max_ was calculated as described in the Materials and Methods section. The data are expressed as the mean ± standard error of the mean (S.E.M.) of three independent experiments performed in triplicate. D_2L_-R, long form of the D_2_ dopamine receptor; IC_50_, the half maximal inhibitory concentration.

Construct	IC_50_ (nM)	B_max_ (Fold Change)
D_2L_-R	0.82 ± 0.17	1.00 ± 0.00
M1	1.84 ± 0.03	2.20 ± 0.54
M2	1.11 ± 0.19	1.84 ± 0.33
M3	1.74 ± 0.29	2.27 ± 0.36
M4	1.26 ± 0.03	2.15 ± 0.77
M5	1.83 ± 0.60	2.94 ± 0.48
M6	2.40 ± 0.02	3.72 ± 1.40
M7	2.93 ± 1.41	3.40 ± 2.03

**Table 2:** Maximal agonist-induced BRET^2^ (BRET_max_) and pharmacological characterization of βarr2/receptor interaction. HEK-293 cells were transiently transfected with GFP^2^/βarr2 R393E,R395E mutant and the indicated RLuc8-tagged receptor construct. Cells were treated with an increasing concentration (10^−11^ to 10^−4^ M) of dopamine or were pretreated for 15 min with an increasing concentration (10^−12^ to 10^−5^ M) of sulpiride before dopamine was added (final concentration 1 μM). BRET_max_ is presented as the fold change relative to D_2L_R-RLuc8 and GFP^2^/βarr2 R393E,R395E-transfected cells. The EC_50_ and IC_50_ values were obtained by sigmoidal dose-response curve fit (GraphPad Prism). The data shown are the mean ± S.E.M. of triplicate observations from 3 to 5 independent experiments. The significance relative to the D_2L_-R was determined by one-way ANOVA with Dunnett’s post hoc test (*, *p* < 0.05). EC_50_, the half-maximal effective concentrations; IC_50_, the half maximal inhibitory concentration.

Construct	BRET_max_ (Fold Change)	EC_50_ (nM)	IC_50_ (nM)
D_2L_-R/RLuc8	1.00 ± 0.10	140.5 ± 31.6	149.7 ± 54.4
M1/RLuc8	1.41 ± 0.03 *	232.1 ± 73.1	99.5 ± 10.1
M2/RLuc8	1.51 ± 0.10 *	222.8 ± 62.2	129.9 ± 16.8
M3/RLuc8	1.44 ± 0.07 *	183.6 ± 28.1	107.1 ± 24.1
M4/RLuc8	1.54 ± 0.09 *	218.3 ± 40.7	98.8 ± 9.1
M5/RLuc8	1.51 ± 0.07 *	208.8 ± 45.4	100.7 ± 37.5
M6/RLuc8	1.41 ± 0.09 *	318.8 ± 145.2	96.1 ± 30.5
M7/RLuc8	1.31 ± 0.05	327.9 ± 119.4	75.8 ± 23.9

**Table 3:** Signaling properties of the D_2L_-R constructs in HEK-293 cells. The half-maximal effective concentrations (EC_50_) for the inhibition of forskolin-stimulated cAMP accumulation were determined for the D_2L_-R/Rluc8 and D_2L_-R/Rluc8 mutants that were transiently expressed in HEK-293 cells using HitHunter^®^ cAMP assay (DiscoveRx Corporation Ltd., Fremont, CA, USA) as described in the Materials and Methods section. The data shown are the mean ± S.E.M. from three independent experiments. The EC_50_ values were obtained by sigmoidal dose response-curve fit (GraphPad Prism). The efficacy of the D_2L_-R constructs in inhibiting forskolin-induced cAMP accumulation is given as the fold change relative to D_2L_-R. The significance relative to the D_2L_-R was determined by one-way ANOVA with Dunnett’s post hoc test (*, *p* < 0.05).

Construct	EC_50_(nM)	Inhibition of Forskolin—Induced cAMP Accumulation (Fold Change)
D_2L_-R/RLuc8	1.88 ± 0.57	1.00 ± 0.04
M1/RLuc8	3.68 ± 0.55	2.09 ± 0.21
M2/RLuc8	2.45 ± 0.87	1.74 ± 0.22
M3/RLuc8	2.82 ± 0.66	2.96 ± 0.35 *
M4/RLuc8	2.17 ± 0.96	2.91 ± 0.48 *
M5/RLuc8	1.15 ± 0.68	4.46 ± 0.81 *
M6/RLuc8	2.82 ± 0.89	2.85 ± 0.59 *
M7/RLuc8	1.91 ± 1.2	1.68 ± 0.22

## Supplementary Materials

Supplementary materials can be found at http://www.mdpi.com/1422-0067/17/7/1152/s1.
