# Supplementary material for: The Conserved Arginine Cluster in the Insert of the Third Cytoplasmic Loop of the Long Form of the D2 Dopamine Receptor (D2L-R) Acts as an Intracellular Retention Signal
Source: Int J Mol Sci. 2016 Jul 19;17(7):1152. doi: 10.3390/ijms17071152 (PMC4964525; doi:10.3390/ijms17071152)
Supplement: Supplementary file 1 [file ijms-17-01152-s001.pdf]

Supplementary Materials: The Conserved Arginine Cluster in the Insert of the Third Cytoplasmic Loop of the Long form of the D_2 Dopamine Receptor (D_{2L}-R) Acts as an Intracellular Retention Signal

Valentina Kubale, Kaja Blagotinšek, Jane Nøhr, Karin A. Eidne and Milka Vrecl

Table S1. The degree of colocalization between the D_{2L}-R constructs and calnexin in transiently transfected HEK-293 cells.

Construct	Rcoloc
D_{2L}-R	0.76 ± 0.03
M1	0.40 ± 0.07 *
M2	0.48 ± 0.10 *
M3	0.51 ± 0.03 *
M4	0.43 ± 0.01 *
M5	0.41 ± 0.04 *
M6	0.42 ± 0.06 *
M7	0.44 ± 0.07 *

*, $p < 0.05$.

Pearson's correlation coefficient for image above thresholds (Rcoloc) was determined as described in Material and Methods. Rcoloc represents pixels where both channels (red and green) were above their respective threshold; its values range between −1.0 and 1.0, where −1, 0 and +1 indicates complete negative correlation, no significant correlation and perfect correlation, respectively. The data shown are the mean ± standard error of the mean (S.E.M.) of five to seven individual cells showing colocalization between the individual D_{2L}-R construct and ER marker calnexin. The significance relative to the D_{2L}-R was determined by one-way ANOVA with Dunnett's post hoc test (*, $p < 0.05$).
